# An Unusual Case of Limbic Encephalitis Caused by Whipple Disease

**DOI:** 10.7759/cureus.65385

**Published:** 2024-07-25

**Authors:** Aimee Miller, Johann C Park, Angela Penney, John J Geraghty, Forshing Lui

**Affiliations:** 1 College of Medicine, California Northstate University, Elk Grove, USA; 2 Department of Neurology, Kaiser Permanente Roseville Medical Center, Roseville, USA

**Keywords:** arthritis, dementia, tropheryma whipplei, neuroimaging, limbic encephalitis, nervous central system, whipple's disease

## Abstract

Whipple disease (WD), a multisystemic infectious disorder caused by *Tropheryma whipplei* (*T. whipplei*), typically presents with gastrointestinal (GI) symptoms such as abdominal pain, diarrhea, GI bleeding, and weight loss. Uncommonly, it can also involve the central nervous system (CNS) and may present with a range of symptoms, including personality changes, dementia, and rhombencephalitis. Prompt antibiotic treatment and careful follow-up are crucial for favorable patient outcomes and a reduction in morbidity and mortality. In this case, we describe a 46-year-old male with primary CNS-WD and discuss the symptomatic manifestations, diagnostic findings, differential diagnosis, and management. This patient initially presented with arthritic complaints and, over a five-year period, developed progressive neurocognitive symptoms, including anxiety, panic attacks, retrograde amnesia, personality changes, aphasia, anhedonia, dysarthria, and rapidly progressive dementia. Magnetic resonance imaging (MRI) revealed symmetric T2 fluid-attenuated inversion recovery (FLAIR) hyperintensities in the bilateral medial temporal lobes, hippocampi, and hypothalamus. A lumbar puncture (LP) showed mild pleocytosis and elevated protein, with no autoimmune or paraneoplastic causes. Temporal lobe biopsy revealed rod-like structures, and *T. whipplei* DNA was confirmed by polymerase chain reaction (PCR). This case underscores the importance of maintaining a high index of suspicion for WD in patients presenting with atypical symptoms with rapidly progressive dementia, as early detection and management are key to circumventing irreversible neurological damage and death.

## Introduction

Whipple disease (WD) is a rare, chronic multisystemic infectious disorder caused by the bacterium *Tropheryma whipplei *(*T. whipplei*). In addition to the rarity of the condition itself, the various symptomatic manifestations of WD have posed a diagnostic challenge since the initial description of the disease by Dr. George Hoyt Whipple in 1907 [1]. The etiology of WD remained unknown until suspicion of an infectious cause arose with significant responsiveness to antibiotic administration in the treatment of diarrhea as a symptom in the 1950s and subsequent identification of the bacillus with electron microscopy in the 1960s [2,3]. This theory of *T. whipplei* as the cause of WD was confirmed more recently in 1991 using polymerase chain reaction (PCR) to determine the 16S rRNA gene sequence of the bacillus, contributing to the facilitation of different possible diagnostic tests [4]. WD is the chronic development of infection with *T. whipplei* and classically presents as a malabsorption disorder with heterogeneous gastrointestinal (GI) manifestations such as abdominal pain, diarrhea, GI bleeding, and weight loss due to host immune-mediated inflammation of the mucosa [5]. The majority of patients also exhibit intermittent fever alongside intermittent migratory seronegative arthritis/arthralgia early in the course of the disease. Consequently, this clinical presentation warrants caution as a misdiagnosis of a rheumatologic disorder and treatment with immunosuppressants can accelerate the trajectory of WD. In addition to these classical symptoms, literature has revealed WD involves a broad range of other organ systems including cardiac, pulmonary, lymphatic, and central nervous system (CNS) [6]. 

Prevalence of CNS manifestations is among the rarest of the non-classical presentations: cerebral involvement is observed in 20-40% of patients with GI-WD, and isolated neurological symptoms in only 4-5% of WD cases [7]. Neurologic-related symptomatology can include progressive dementia, mood alteration, ophthalmoplegia, hypothalamic dysfunction (appetite/sleep-cycle disorders), myoclonus, and encephalitis [8]. Neurologic symptoms in WD foreshadow a poor prognosis, and the low incidence of CNS involvement alongside the atypical nature of this challenging diagnosis has limited current literature. Nonetheless, these presentations are important to consider and treat before neurologic sequelae become irreversible, as mortality risk is significant for this otherwise treatable disease. Early detection of WD relies on a myriad of tests including PCR analysis of cerebrospinal fluid (CSF), magnetic resonance imaging (MRI) of the brain or other clinically relevant areas, and a brain biopsy if warranted by strong suspicion despite negative tests [9]. While untreated WD proves to be fatal, prompt treatment with antibiotics and attentive follow-up to prevent relapse are critical for a favorable outcome. Considering the relatively small number of cases reported on primary CNS-WD, each patient with this presentation acts as a remarkable step forward in our understanding of how to better detect, diagnose, and treat this disease.

In this paper, we report the case of WD in a patient with prominent neurologic presentation. Informed consent was obtained from the patient, and IRB is exempt as all patient information has been de-identified. Neurologic manifestations of memory loss, psychiatric symptoms, and sleep changes developed after initial follow-ups on arthritis and GI symptoms. Despite an early negative duodenum biopsy for GI WD, a later brain biopsy and positive periodic acid-Schiff (PAS) stain confirmed the diagnosis for CNS-WD.

## Case presentation

A 46-year-old male initially reported seronegative polyarthritic symptoms, which were managed and followed by rheumatology in 2016. In 2018, the patient began to manifest with anxiety and panic attacks, with no prior episodes noted. In June 2020, the patient presented with new onset memory loss, where he was unable to recall major life events, and in July 2020, he presented with psychiatric issues including new-onset depression and worsening anxiety and panic attacks. During this time period, he also reported somnolence and noted that his sensitivity to light touch seemed to be diminished. Following this, in October 2020, he was noted to have more significant cognitive symptoms, including decreased comprehension, loss of personality, anhedonia, slurring of speech and mumbling, and a general loss of independence due to the inability to attend to activities of daily living. 

**Figure 1:**
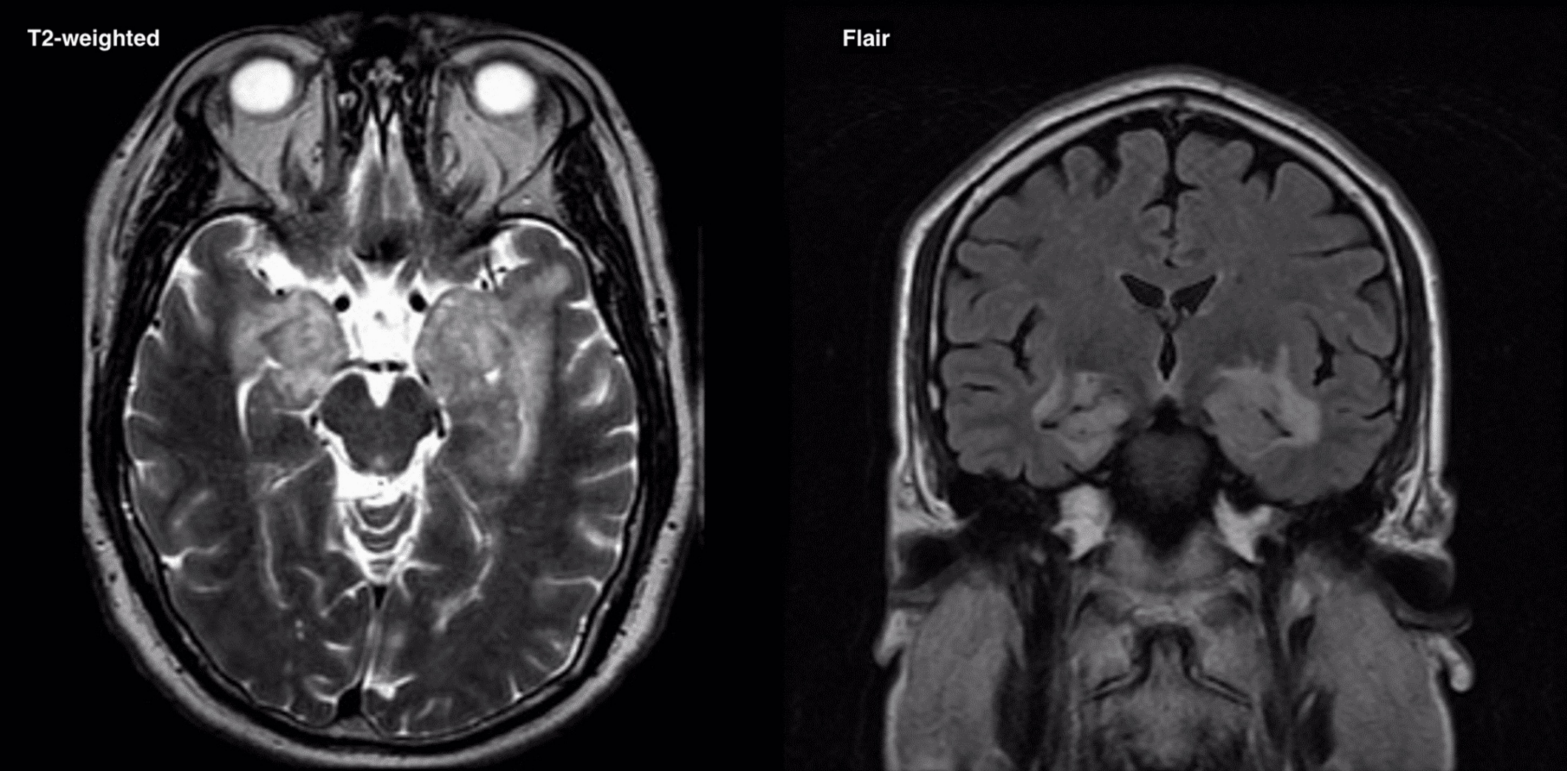
T2-weighted (left) and FLAIR MRI (right) Demonstrating relatively symmetric hyperintensities affecting both medial temporal lobes, involving the hippocampi, amygdala, hypothalamic, and optic chiasma FLAIR: Fluid-attenuated inversion recovery; MRI: Magnetic resonance imaging

**Figure 2:**
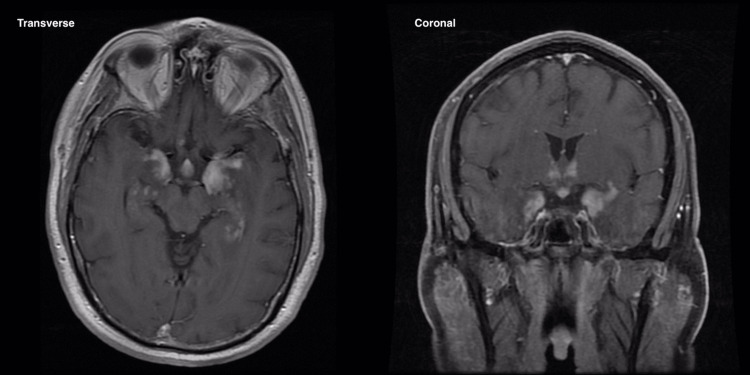
Transverse (left) and coronal T1-weighted MRI (right) Showing further contrast enhancement of the lesions MRI: Magnetic resonance imaging

Beginning in January 2021, the patient was seen by neurology for further evaluation of his rapidly declining cognitive function. Due to his neurological manifestations, the patient underwent multiple investigations, including chest computerized tomography (CT), brain MRI, and a lumbar puncture (LP). The chest CT was negative, while the MRI showed relatively symmetric T2 fluid-attenuated inversion recovery (FLAIR) hyperintensities with enhancements in both medial temporal lobes and hippocampi, and the hypothalamus (Figure 1). T1-weighted MRI showed similar contrast enhancement of the hippocampi and the hypothalamus (Figure 2). The LP showed mild pleocytosis with a white blood cell (WBC) count of 47/µL with 87% lymphocytes and an elevated protein of 103 mg/dL. Based on these results, a diagnosis of limbic encephalitis (LE) was made. The CSF was further analyzed at the Mayo Clinic for an autoimmune panel and paraneoplastic panel, both of which were negative. The patient subsequently was treated empirically for acute LE with plasmapheresis and intravenous (IV) and oral steroids, with which he showed some improvement. Further workup was performed and included a repeat LP, a left temporal lobe biopsy, and a duodenal biopsy. The repeat LP showed a WBC count of 13/µL with 96% lymphocytes and an elevated protein of 57 mg/dL. Histological analysis of the temporal lobe biopsy revealed reactive gliosis with chronic inflammation consisting of lymphocytes and amalgamated histiocytes with abundant gray cytoplasm and atypical granules within the cytoplasm. Further histology analyses revealed intracytoplasmic and extracellular rod-like structures detected with PAS and Grocott methenamine silver (GMS) staining techniques. A diagnosis of WD was confirmed by PCR due to the detection of *T. whipplei* DNA in a brain tissue sample. No histological features associated with WD were detected from the duodenal biopsy. 

## Discussion

The clinical presentation of CNS-WD includes a wide variety of symptoms. Limbic-predominant involvement more commonly includes psychiatric symptoms, such as new or worsening anxiety or depression, and acute to chronic cognitive decline [10]. Patients may also experience hypothalamic symptoms related to sleep disturbances, polydipsia, and hyperphagia [10]. Alternatively, rhombencephalitis is another unique presentation of CNS-WD that may appear as hemiparesis, hypesthesia, vertigo, headache, or diplopia. Oculomasticatory myorhythmia, an ocular movement disorder characterized by rhythmic contractions of the oromandibular and ocular muscles, is an uncommon yet highly specific finding for this subset of CNS-WD [11]. Collectively, symptoms of dementia and oculomasticatory myorhythmia should increase clinical suspicion for a CNS-WD diagnosis [10].

Our patient presented with rather typical clinical and imaging features of LE, including memory loss, anxiety, and depression, without any evidence of rhombencephalitis. LE is not commonly caused by bacterial infections, such as *T. whipplei*, with the notable exception of neurosyphilis; it is more frequently associated with autoimmune processes, paraneoplastic syndromes, and viral infections [12]. Autoimmune LE classically involves altered consciousness and psychiatric clinical features. Studies have found that approximately 50% of autoimmune LE cases have abnormal MRI findings, and inflammatory damage is often not limited to the limbic region [13]. In contrast, herpetic encephalitis classically presents with fever, altered consciousness, memory impairment, and MRI demonstrating abnormal signals in the hippocampi and amygdala, but without basal ganglia involvement [14]. Radiologically, CNS-WD does not follow a definite pattern, with some patients’ MRIs showing focal lesions in the hypothalamus, temporal lobes, hippocampus, pons, medulla, or cerebellum [15-17], while other patients’ MRIs demonstrate normal radiological patterns [18]. In regard to CSF findings, both autoimmune LE and CNS-WD display CSF pleocytosis >5 WBC/µL, but CNS-WD can be differentiated from autoimmune LE by an elevated protein count in the CSF >45 mg/dL [19].

Crucial for diagnosis in our patient was a left temporal lobe biopsy showing a positive PAS stain, consistent with CNS-WD, following a negative duodenal biopsy. The diagnosis was then confirmed by PCR with the detection of *T. whipplei* DNA. Combined PCR tests and PAS-stained biopsies are imperative for proper diagnosis of CNS-WD, as biopsies alone have a higher probability of false-positive results [20].

## Conclusions

In this report, we have presented a case of primary CNS-WD in a middle-aged patient with rapidly progressive dementia. CNS-WD occurs with a wide variety of presentations, and the non-specificity of its clinical features can make it a particularly challenging diagnosis to make. While primarily affecting the GI system, it can also lead to an array of neurological symptoms, most notably LE. Patients with LE may experience memory issues, personality changes, and cognitive deficits, but the manifestations can also extend to seizures, psychosis, and various other neurological deficits. This spectrum of symptoms for LE is prevalent across numerous other neurologic and neuropsychiatric disorders, emphasizing the complexity of diagnosing and treating primary CNS-WD that presents as LE. The unique presentation of this case of primary CNS-WD with LE, which lacks evidence of rhombencephalitis, indicates the importance of a high suspicion index in the diagnostic approach. Hence, for optimized earlier detection and effective treatment, clinicians should remain vigilant and suspicious of primary CNS-WD when encountering similar encephalopathic manifestations.
